# Immunological and Inflammatory Impact of Non-Intubated Lung Metastasectomy

**DOI:** 10.3390/ijms18071466

**Published:** 2017-07-07

**Authors:** Tommaso Claudio Mineo, Francesco Sellitri, Gianluca Vanni, Filippo Tommaso Gallina, Vincenzo Ambrogi

**Affiliations:** 1 Department of Surgery and Experimental Medicine, Tor Vergata University of Rome, Rome 00173, Italy; fsellitri68@gmail.com (F.S.); vanni_gianluca@yahoo.it (G.V.); filippogallina92@gmail.com (F.T.G.); ambrogi@med.uniroma2.it (V.A.); 2 Department of Thoracic Surgery, Official Awake Thoracic Surgery Research Group, Policlinico Tor Vergata University of Rome, Roma 00133, Italy

**Keywords:** lung metastases, non-intubated surgery, video-assisted thoracic surgery

## Abstract

Background: We hypothesized that video-assisted thoracic surgery (VATS) lung metastasectomy under non-intubated anesthesia may have a lesser immunological and inflammatory impact than the same procedure under general anesthesia. Methods: Between December 2005 and October 2015, 55 patients with pulmonary oligometastases (at the first episode) successfully underwent VATS metastasectomy under non-intubated anesthesia. Lymphocytes subpopulation and interleukins 6 and 10 were measured at different intervals and matched with a control group composed of 13 patients with similar clinical features who refused non-intubated surgery. Results: The non-intubated group demonstrated a lesser reduction of natural killer lymphocytes at 7 days from the procedure (*p* = 0.04) compared to control. Furthermore, the group revealed a lesser spillage of interleukin 6 after 1 (*p* = 0.03), 7 (*p* = 0.04), and 14 (*p* = 0.05) days. There was no mortality in any groups. Major morbidity rate was significantly higher in the general anesthesia group 3 (5%) vs. 3 (23%) (*p* = 0.04). The median hospital stay was 3.0 vs. 3.7 (*p* = 0.033) days, the estimated costs with the non-intubated procedure was significantly lower, even excluding the hospital stay. Conclusions: VATS lung metastasectomy in non-intubated anesthesia had significantly lesser impact on both immunological and inflammatory response compared to traditional procedure in intubated general anesthesia.

## 1. Introduction

The increasing evolution of non-intubated thoracic surgery allowed the execution of progressively more complicated operations in patients with different pathologies [1,2,3,4,5,6]. Our program of non-intubated thoracic surgery named the Awake Thoracic Surgery Research Group is—to our knowledge—the oldest surgical program specifically created for this purpose by one of us (TCM), who is still the main coordinator [7]. To date, more than one thousand non-intubated procedures were carried out in our department [8]. Surgery of lung metastases has been performed since the beginning of our experience [9]. Early operations were done under epidural anesthesia and three-port video-assisted thoracic surgery (VATS) [10] but starting from 2005, lung metastasectomies have been preferably accomplished through a unique thoracoscopic access under non-intubated anesthesia [11].

Traditional intubated surgery [12,13] and moreover one-lung ventilation [14,15,16] demonstrated several important adverse effects in both systemic inflammation and immunology, thus facilitating postoperative infections and cancer recurrence [17,18,19]. Conversely, the effects of non-intubated operations have been extensively evaluated over the years, disclosing intriguing implications on inflammatory stress [20] and immunological response [21]. As a matter of fact, these operations proved capable of generating a lower level of inflammation and lesser degree of immunologic depression than the traditional ones [22,23]. On these bases, we think that the use of non-intubated anesthesia appears particularly suitable in the surgery of oligometastatic patients. Herein, we analyzed some pattern of inflammatory and immunological response after lung metastasectomy carried out under non-intubated anesthesia.

## 2. Results

Demographic and pathological features of the two groups resulted homogeneous, as shown in Table 1.

### 2.1. Immunological Impact

**Figure 1:**
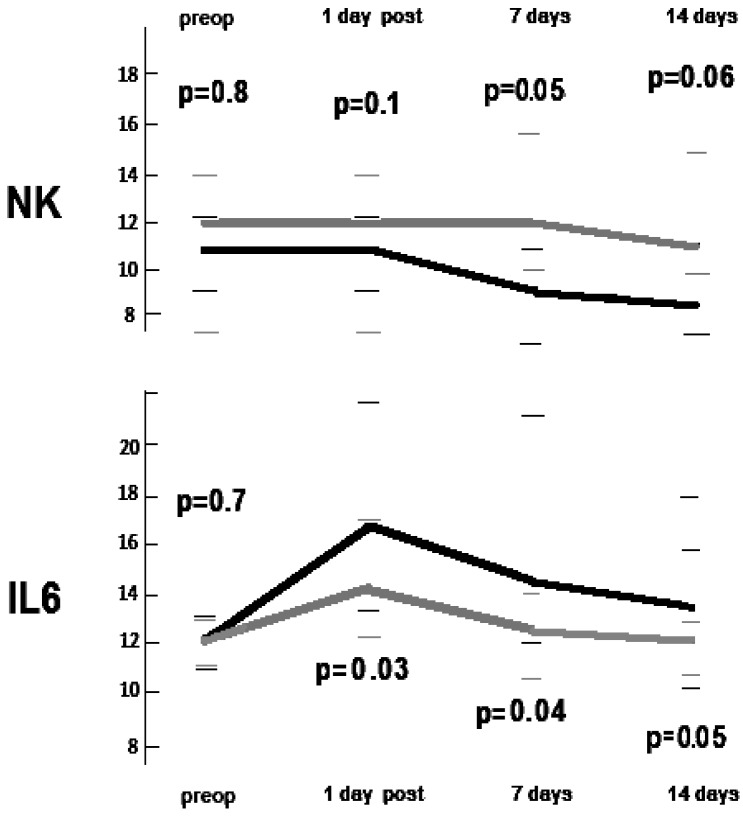
Median postoperative changes of natural killer (NK) lymphocytes and interleukin-6 (IL-6) for intubated (black line) and non-intubated (gray line) patients. Between group *p*-values at different intervals and interquartile ranges are indicated.

**Figure 2:**
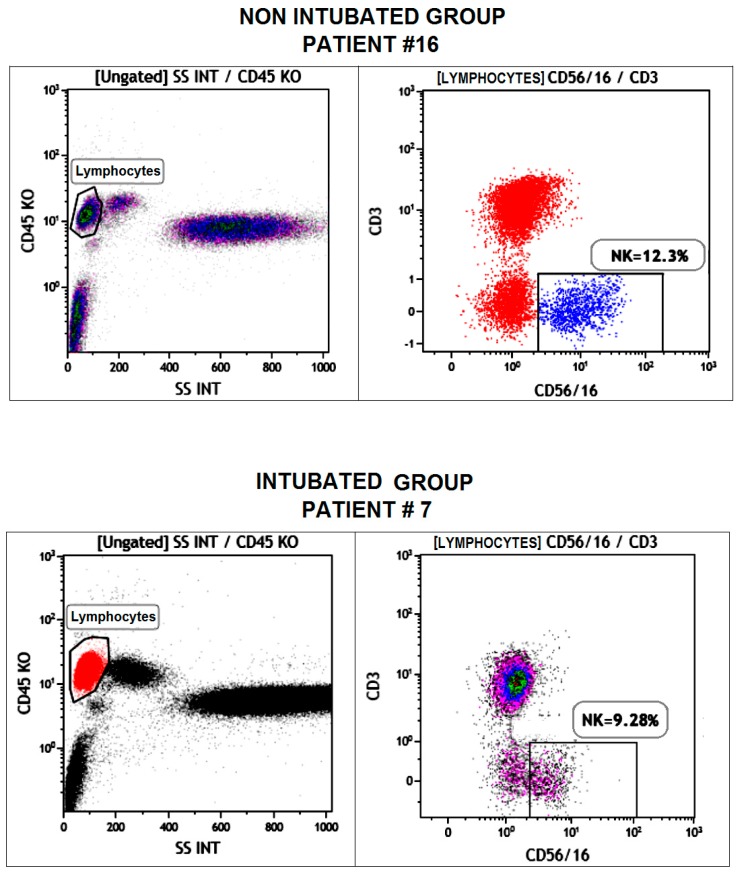
Dot-plot flow cytometry photos from samples withdrawn after 7 days post-operation in two different and representative patients belonging to the non-intubated (**top**) and intubated (**bottom**) groups and demonstrating the higher level of natural killer cells in the non-intubated patient.

Postoperative immunologic trends are shown in Table 2 and in Figure 1. A representative fluorescence-activated cell sorting (FACS) photo is shown in Figure 2. As expected, total leukocytes count increased after surgery in both groups. However, we found a more rapid decrement in the non-intubated group but without reaching the between-group significance threshold (*p* = 0.06). The total lymphocytes count showed a lesser drop in the non-intubated group in both post-operative day 1 (*p* = 0.05) and post-operative day 7 (*p* = 0.05), with the non-intubated group also displaying a nearly-significant more rapid restoration of the baseline value. Among the subpopulations in the non-intubated group, there was a significant lesser reduction of natural killer lymphocytes at 7 days following the procedure (*p* = 0.04) compared to the intubated group (Figure 1). On the other hand, the other subpopulations did not present significant difference between groups.

### 2.2. Inflammatory Impact

The postoperative variations of interleukin 6 and interleukin 10 are reported in Table 3. As expected, the values increased rapidly in the postoperative period, persisting above the baseline values for the whole observation period. However, interleukin 6 showed a more significant increment in the intubated group starting from day 1 (between-group difference *p* = 0.03) and persisting at day 7 (*p* = 0.04) and day 14 (*p* = 0.05) (Figure 1). No differences between groups were found in interleukin 10 levels.

### 2.3. Morbidity

There was neither in-hospital nor 30-day postoperative mortality in both groups. Major morbidity rate was significantly higher in the intubated group 3 (5%) vs. 3 (23%) (*p* = 0.04). In the non-intubated group, we experienced only two patients with persistent air leak and one with arrhythmia, whereas in the intubated group two patients developed pneumonia and one had a persistent air leakage.

The median hospital stay was 3.0 vs. 3.7 days (*p* = 0.033), but even excluding the hospital stay, the estimated costs for the non-intubated procedures were significantly lower (median expenses: €3100 vs. €3900; *p* = 0.03).

## 3. Discussion

Morbidity rate after thoracic surgery is often related to one-lung ventilation [14,15,16,24], although mitigated by the minimally invasive approaches [25,26]. In particular, there is increasing evidence that one-lung ventilation might generate a number of anatomic changes in both dependent and non-dependent lungs. Their effects are similar to a compartmental inflammatory injury [27,28,29,30,31,32,33] that may impact the immunological response.

In the present study, we found that the non-intubated procedure can reach successful results with a significantly lower morbidity rate. The exiguous number of intubated patients did not allow strong conclusions to be drawn. However, we experienced a significantly lower decrement of natural killer lymphocytes at day 7 as well as a significant attenuation of interleukin 6 response. Avoidance of one-lung ventilation may also have contributed to the more physiologic lymphocyte response observed in non-intubated patients. The effects of one-lung ventilation on natural killer activity have been known since 1993 [34]. Furthermore, other authors [35,36,37] have shown that one-lung ventilation can evoke a cascade of many oxidative changes, eventually resulting in a compartmental release of pro-inflammatory mediators including interleukin 6. The activation and secretion of this mediator could lead to a transient increase of cortisol plasma level, interfering with natural killer activity [38,39].

This immune-depressive effect induced by one-lung ventilation may also have an impact on oncological conditions. It is not rare that patients operated for lung metastases rapidly develop an unexpected new lung metastasis [18]. This may be due to the presence of occult metastases that had a rapid growth to the lack of immune control related to postoperative immunologic depression [40,41,42]. In our previous study, we did not find significant differences in postoperative survival in patients undergoing colorectal lung metastasectomy [11], but a larger study sample with longer follow up and hopefully on a randomized basis will probably achieve different results.

The surgery of lung metastases is an argument that has always stimulated our attention [43,44,45,46,47]. Since 2000, we started a program of VATS operations under thoracic epidural anesthesia in awake and collaborative patients affected from different pathologies [8]. To our knowledge, this is the oldest surgical program specifically created for this purpose. The confidence in this kind of procedure is now quite high and increasingly recognized all over the world. Despite the surgical pneumothorax, the evaluation of vital parameters showed a satisfactory arterial oxygenation both intra and postoperatively [11]. This allowed an immediate resumption of many daily activities, faster recovery, shorter hospitalization and lower economical costs. The further data presented in this paper about inflammatory and immunological response may contribute to the justification of a rationale for lower morbidity and increase the confidence in this kind of procedure.

We acknowledge that this study has evident limitations due to its non-randomized nature and small control group. However, we think of this as an observational study prior to reaching a more robust evidence through more structured and controlled investigations.

## 4. Materials and Methods

Between December 2004 and October 2015, a total of 55 patients referred to our center for pulmonary oligometastases successfully underwent uniportal VATS lung metastasectomy under non-intubated anesthesia. Clinical features of the patients cohort in the study are summarized in Table 1. Thirteen patients scheduled in the same period for the same procedure who refused the non-intubated anesthesia were used as a control group. They underwent a traditional VATS procedure in general anesthesia under one-lung ventilation.

The study was a single-center and retrospective matched analysis between a non-intubated group vs. control group undergoing metastasectomy under intubated general anesthesia. Inclusion criteria for the non-intubated surgery were patient’s preference, generic indications to non-intubated anesthesia [9], and the presence of peripheral oligometastases—no more than two—at the first episode and resectable with a wedge resection. Bilateral lesions were approached in two separate sessions in different days. This study was submitted and approved by the Internal Review Board at Tor Vergata University of Rome with the authorization code 627/15.

Electrocardiogram, pulse oximeter, systemic and central venous blood pressure, body temperature, arterial blood gases, end-tidal CO_2_, and bispectral index were continuously monitored during the operation [48]. Just before the procedure, a 5 mL solution of 2% lidocaine was aerosolized for 5 min to prevent cough reflex. During the operation, the patient inhaled O_2_ through a ventimask to maintain saturation greater than 90%. Intercostal bloc was habitually achieved by separate local injection of lidocaine 2% (4 mg/kg) and ropivacaine 7.5% (2 mg/kg). All intrathoracic phases were regularly well tolerated by intraoperative intravenous administration of benzodiazepine (midazolam 0.03–0.1 mg/kg) or opioids (remifentanil 15 μg/kg/min). Incidental anxiety or a panic occurring intraoperatively were sedated slightly by increasing the continuous propofol (0.5 mg/kg) infusion without interfering with spontaneous breathing.

The procedures were accomplished with the patient lying in lateral decubitus position through a single small 30–40 mm port incision located at the most fitting intercostal space to reach and remove the suspect nodule. Intercostal muscles were retracted by the Alexis (Alexis^®^, Applied Medical, Rancho Santa Margarita, CA, USA), thus allowing the introduction of the thoracoscope and the instruments. Whenever necessary, a mounted gauze pad to hinder pulmonary movements was also introduced. The lesion was detected by both digital and instrumental palpation and resected by linear stapler. At the end of the procedure, one 28 Ch chest tube was collocated at the posterior limit of the surgical wound. Drinking, eating, and walking was generally allowed in the same day of surgery. Patients were discharged after radiological evidence of complete lung re-expansion, limited pleural effusion (no more than 100 mL/day), and no air leakage. Patients with protracted air leakage (>5 days) were discharged with a Heimlich valve.

Blood samples were always withdrawn through an antecubital vein in the morning (7:30 a.m.) just prior the operating session and at postoperative days 1, 7, and 14. Samples were sent to the Laboratory of Onco-hematology of our institution for immediate real-time tests without need of storage. Total lymphocytes were measured with a cell counter (Coulter Beckmann, MedLab, Cupertino, CA, USA).

For lymphocyte-subset assessment, the blood samples were incubated for 30 min with monoclonal antibodies at 4 °C. The samples were processed with a coulter, which lyses the erythrocytes, and stabilizes and fixes the leukocytes. Lymphocyte-subset were acquired and analyzed by FACSCanto II esa-color flow cytometry (BD Biosciences, San Diego, CA, USA) with antibodies specific to the cell markers. Samples were incubated with monoclonal antibodies and then processed with the lyse-wash technique (ammonium-chloride solution 1×; BD, Biosciences). Phenotypes of lymphocyte population were identified by anti-cluster of differentiation 3-fluorescein isothiocyanate (anti-CD3-FITC), anti-cluster of differentiation 4-allophycocyanin-H7 (anti-CD4-APC-H7), anti-cluster of differentiation 8-R-phycoerythrin-cyanine 7 (anti-CD8-PE-Cy7), anti-CD56(3-)-PE, anti-CD19-APC, and anti-cluster of differentiation 45-peridinin chlorophyll/cyanine 5.5 (anti-CD45-PercP/Cy5.5) (BD Biosciences, San Diego, CA, USA).

Circulating concentrations of interleukin 6 and 10 were measured using commercially available human colorimetric enzyme-linked immunosorbent assays (Quantikine ELISA, R & D Systems, Europe Ltd., Abingdon, UK).

Statistical analysis was performed with the SPSS 18 computer software package (SPSS^®^ 18 version, Chicago, IL, USA). Non-parametric tests were prudentially preferred using Wilcoxon for within group and Kruskal–Wallis for between-group evaluations, respectively. Data were expressed as median interquartile range. Significant threshold was considered *p* < 0.05.

## 5. Conclusions

In the last decades, increasing attention has been dedicated to the importance of systemic inflammation and immune-competence in oncologic patients. Uniportal VATS lung metastasectomy in non-intubated anesthesia had a significant lower impact on both immunological and inflammatory response compared to the traditional procedure in general anesthesia, intubation, and one-lung-ventilation.

## Figures and Tables

**Table 1:** Biological features of the two groups.

	Non-Intubated Group (*n* = 55)	Intubated Group (*n* = 13)	*p*-Value
Age (range), years	64 (47–74)	66 (51–73)	0.1
Sex (m:f)	30:25	7:6	0.9
**Primitive Histology**			
carcinoma:sarcoma	45:10	13:0	0.09
**Previous Adjuvant Chemotherapy**			
yes:no	37:18	9:4	0.8
**Disease-Free Interval**			
<1 year	28	6	0.7
>3 years	16	3	0.6
Resected metastases (mean)	1.55	1.77	0.8

**Table 2:** Postoperative changes of lymphocyte subpopulations. Interquartile ranges are expressed within brackets.

	Baseline	Day 1	Between-Group *p*-Value	Day 7	Between-Group *p*-Value	Day 14	Between-Group *p*-Value
**Total Leucocytes (*n* 10^9^/L)**							
*Non-intubated group*	5.45 (4.22–8.44)	7.51 (5.15–9.02)	0.08	6.04 (5.36–9.32)	0.06	6.17 (5.07–8.73)	0.3
*Intubated group*	5.81 (4.47–8.21)	8.01 (7.44–9.82)		7.33 (7.06–9.98)		6.34 (5.32–8.41)	
**Total Lymphocytes (*n* 10^9^/L)**							
*Non-intubated group*	1.91 (1.67–2.45)	1.86 (1.52–1.99)	0.05	1.89 (1.53–2.12)	0.05	1.91 (1.03–2.09)	0.06
*Intubated group*	1.90 (1.53–2.39)	1.69 (1.46–2.05)		1.69 (1.29–1.91)		1.71 (1.21–2.04)	
**B Lymphocytes (%)**							
*Non-intubated group*	11 (7–15)	12 (7–16)	0. 8	11 (7–15)	1	9 (7–16)	0.9
*Intubated group*	12 (7–15)	12 (7–15)		11 (7–15)		9 (7–15)	
**T Lymphocytes (%)**							
*Non-intubated group*	71 (60 –75)	70 (64–73)	0.1	73 (65–85)	0.06	73 (61–77)	0.3
*Intubated group*	72 (64–75)	67 (61–76)		64 (61–75)		70 (69–86)	
**T Helper/T Suppressor (Ratio)**							
*Non-intubated group*	2.3 (1.3–3.7)	2.3 (1.3–3.3)	0.9	2.3 (2.0–3.9)	0.9	2.3 (1.7–3.6)	0.9
*Intubated group*	2.3 (1.3–3.5)	2.3 (1.3–3.1)		2.3 (1.7–3.8)		2.1 (1.6–2.8)	
**Natural-Killer (%)**							
*Non-intubated group*	12 (7–14)	12 (7–14)	0.09	12 (10–16)	0.04	11 (10–15)	0.06
*Intubated group*	11 (9–12)	11 (9–12)		9 (7–11)		9 (8–16)	

**Table 3:** Postoperative changes of cytokines. Interquartile ranges are expressed within brackets.

	Baseline	Day 1	Between-Group *p*-Value	Day 7	Between-Group *p*-Value	Day 14	Between-Group *p*-Value
**IL–6 (pg/mL)**							
*Non–intubated group*	12.1 (11.1–12.9)	14.1 (11.9–17.3)	0.03	12.5 (10.4–14.4)	0.04	12.0 (10.9–13.1)	0.05
*Intubated group*	12.3 (10.9–13.1)	17.2 (13.3–22.1)		14.5 (12.2–21.1)		13.7 (10.1–18.3)	
**IL–10 (pg/mL)**							
*Non–intubated group*	5.8 (4.3–12.6)	8.4 (5.6–14.1)	0.1	9.6 (9.1–12.2)	0.3	8.1 (7.3–11.1)	0.5
*Intubated group*	5.8 (3.9–12.9)	6.9 (4.2–14.7)		10.9 (7.9–14.2)		9.3 (7.2–13.7)	
